# Recent advances in synthesis and characterization of iron–nickel bimetallic nanoparticles and their applications as photo-catalyst and fuel additive

**DOI:** 10.1039/d3ra04293f

**Published:** 2023-10-10

**Authors:** Saba Jamil, Shanza Rauf Khan, Shamsa Bibi, Nazish Jahan, Nadia Mushtaq, Faisal Rafaqat, Rais Ahmad Khan, Waqas Amber Gill, Muhammad Ramzan Saeed Ashraf Janjua

**Affiliations:** a Super LFight Materials and Nanotechnology Laboratory, Department of Chemistry, University of Agriculture Faisalabad 38000 Pakistan; b Department of Chemistry, College of Science, King Saud University Riyadh 11451 Saudi Arabia; c Departamento de Química Física, Universidad de Valencia Avda Dr Moliner, 50, E-46100 Burjassot Valencia Spain; d Department of Chemistry, Government College University Faisalabad Faisalabad 38000 Pakistan Janjua@gcuf.edu.pk Dr_Janjua2010@yahoo.com +92 300 660 4948

## Abstract

Iron–nickel bimetallic nanoparticles (Fe–Ni BMNPs) are prepared by combining two different metals by using the bottom-up approach. The resulting material has entirely different properties as compared to both the metals. The product is examined by using different analytical instruments such as.; scanning electron microscope (SEM), transmission electron microscope (TEM), X-ray diffraction (XRD), MDIJADE, ORIGIN pro to characterize their morphology, crystallinity and elemental composition and the final data has been statistically analyzed. SEM findings show that most nanoparticles are irregular in form and range in size from 10 nm to 100 nm. The findings of the TEM verified that the particles between 10 nm and 50 nm are irregular in size shape. The products acquired utilized as a fuel additive to monitor oil effectiveness by studying various parameters. The degradation of methylene blue dye depends directly on the concentration of the nanocatalyst. Different parameters also use the freshly prepared bimetallic nanocatalyst to investigate the efficacy of the kerosene fuel. By adding a tiny quantity of the nanocatalyst, the value of the flash point and fire point is significantly reduced. The nanocatalyst does not affect the cloud point and pour point to a large extent. The bimetallic nanocatalyst therefore has very excellent catalytic characteristics.

## Abstract 2

Cubic structure of Fe/Ni bimetallic nanoparticles for their applications as catalyst and fuel additive.

## Graphical abstract

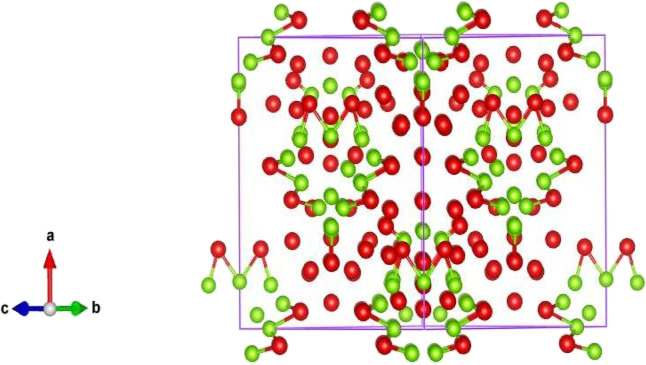

## Introduction

The issue of the growing population and the pollution caused by thermal power stations and industrial units has created significant problems for humans in recent years, as noted by.^1^ However, in the era of nanoscience, the use of nanoparticles (NPs) has the potential to help mitigate these problems due to their excellent thermal stability, catalytic properties, and intrinsic reactivity, according to^2,3^. NPs are particularly interesting in the field of catalysis because they possess dual catalytic properties, as highlighted by.^4^ To produce metal NPs, bulk metals or metal precursors can be converted using either top-down or bottom-up approaches.^5–7^ Top-down methods involve sizing down bulk materials to NP size ranges through approaches like ball milling or attrition, which often result in a wide size distribution, non-uniform shapes, and increased levels of contamination.^8^ On the other hand, bottom-up approaches, such as colloidal synthesis, sol–gel processing, chemical reduction and precipitation, and atomic layer deposition, result in smaller and more uniform sizes and shapes, as well as better control over defects and surface properties, as indicated by.^9,10^ The bottom-up approach to nanoparticle synthesis starts at the atomic or molecular level and gradually progresses to the nanoscale. It involves precise control and manipulation of chemical reactions to produce nanoparticles with specific sizes, shapes, and compositions. This method offers the flexibility to incorporate different functional groups and surface modifications during synthesis, enabling the design of nanoparticles with multiple functionalities to tackle interdisciplinary challenges.^11^ The bottom-up approach can also be scaled up for mass production, making it valuable for industrial applications and commercializing nanoparticle-based products. Additionally, some bottom-up synthesis methods are more environmentally friendly and sustainable compared to top-down approaches. However, there are some limitations to the bottom-up approach, such as the complexity of certain synthesis routes requiring advanced equipment and expertise.^12^ Multi-step processes may result in higher production costs, and some methods can be time-consuming, particularly when creating nanoparticles with intricate structures or precise control over various parameters.^13^

Metallic nanomaterials can be found in monometallic or bimetallic formulations, with the latter often exhibiting more intriguing properties than the former due to synergistic interactions between the two metal.^14,15^ To achieve specific properties and performance, the proper metal combination and support, as well as the composition of each metal type, must be optimized. Bimetallic nanostructures can be classified into two categories: mixed and segregated.^16,17^ Mixed structures can further be categorized into alloyed and intermetallic particles, with the former possessing a random arrangement of atoms and the latter an ordered arrangement.^18^ On the other hand, segregated structures are obtained through multistep reactions, where the first metal type is formed initially, followed by the addition of the second metal.^19,20^ It is worth noting that these structures can be further categorized into several types, including subcluster structures, which feature a separate distribution of two metals with a shared interface; core–shell structures, where a metal core is surrounded by a shell of a second metal; multi-shell core–shell structures, which feature shells with an alternative arrangement that forms a shape like onion rings; and multiple core materials coated by a single shell.^21,22^ These structures are typically synthesized through the concurrent reduction of two metal ions, utilizing appropriate stabilization strategies that enable steric hindrance and electrostatic repulsive forces.^23^ The reduction rates of the two metal precursor ingredients can be controlled during synthesis to achieve the desired size, shape, structure, morphology, and metal distribution of the resulting bimetallic nanoparticles.^24,25^ Various physical and chemical methods can be utilized to synthesize bimetallic nanoparticles, including deposition, co-precipitation, micro-emulsion, hydrothermal, hydrogen plasma reaction, sonochemical, and solvothermal methods.^26,27^ Bimetallic nanoparticles are of great importance in fields such as electronics, photocatalysis, and metallic coating.^28^ Changing the ratio of the different metals used in their synthesis alters the shape of these nanoparticles, while modifying the temperature increases the coordination number of the different atoms, thus enhancing their active sites and catalytic activity.^29^ Due to their inert nature and environmental friendliness, bimetallic nanoparticles are versatile in nature and have various applications in the fields of sensors, electronics, solar cells, and thermometric equipment.^30,31^

Likewise, the majority of catalysts that are sensitive to light have a broad band gap, which allows them to absorb photons with higher energies, particularly in the ultraviolet (UV) region.^32,33^ The production of nanocrystalline bimetallic nanoparticles with large surface areas is challenging, as the solid-state approach requires high temperatures and extended heating times. Nonetheless, bimetallic nanoparticles of this type are in high demand for various applications, including catalysis, environmental cleanup, and solar energy.^34,35^ Nanomaterials have played a significant role in addressing challenges related to air pollution, power shortages, and wastewater treatment, thanks to their smaller size (ranging from 1 to 100 nm) and increased surface area. Bimetallic nanoparticles have demonstrated superior properties compared to monometallic nanoparticles in various technologies.^36–38^

Nowadays, nanoparticles are being employed as a solution for energy crises, domestic and industrial wastewater treatment, and air pollution, among other serious problems.^39,40^ These particles are utilized in various applications, thanks to their specific size and properties, which enhance the characteristics of final products^41^ Morphologically controlled synthesis, characterization and application of zinc–aluminum layered double hydroxide nano needles.^42,43^ This research seeks to prepare nanoparticles, investigate their properties, and develop methods to apply them at a large scale. Specifically, the study focuses on synthesizing bi-metallic particles and utilizing straightforward and innovative techniques to enhance the optical, electrical, and mechanical properties of the resulting products.^44,45^ The bottom-up approach utilized for synthesizing iron–nickel bimetallic nanoparticles involved a systematic procedure that started with high-purity iron and nickel precursors.^46^ These precursors, such as iron chloride (FeCl_3_) and nickel chloride (NiCl_2_), were dissolved in an organic solvent like ethylene glycol or oleylamine, along with the use of surfactants like oleic acid or polyvinylpyrrolidone (PVP) to prevent agglomeration and control particle size.^47^ The precursor solution was then degassed under an inert gas atmosphere to eliminate dissolved gases. Subsequently, the solution was heated and stirred to create a homogenous reaction environment. The reduction reaction took place by adding a suitable reducing agent like hydrazine (N_2_H_4_) or sodium borohydride (NaBH_4_). Throughout the reaction, the temperature and stirring rate were closely monitored and adjusted for optimization.^48^ Discuss the key findings from SEM, TEM, XRD, MDIJ ADE, and ORIGIN pro analyses in separate sections and emphasize their significance in understanding the nanoparticles' structure, morphology, and composition.^49^ The Molecular Dynamics Ionization-Jump Activation Energy (MDIJ ADE) technique was employed to explore the energy required for ionization and ion activation, offering valuable insights into the electronic properties of the nanoparticles. The data obtained from this technique revealed that the iron–nickel bimetallic nanoparticles (Fe–Ni BMNPs) possessed favorable electronic properties suitable for catalytic applications. Additionally, data analysis using ORIGIN Pro software facilitated the determination of various nanoparticle properties, including particle size distribution, crystallinity, and catalytic activity.^50^ The results obtained from ORIGIN Pro demonstrated that the Fe–Ni BMNPs exhibited superior catalytic activity when compared to individual iron or nickel nanoparticles. For the characterization analysis origin pro helped to analyze the graphical data.^51,52^ The product's potential applications have been evaluated using various methods, and the results are presented in both graphical and statistical formats.

## Materials and methods

All the tests needed for this entire study will be performed at the Faisalabad Agriculture University's physical laboratory department of chemistry. Some tests will also be carried out in the other university laboratories.

### Chemicals and reagents

All the chemicals and reagents that are used in this whole research work for the preparation of the bimetallic Fe/Ni nanoparticles are nickel nitrate, ferric chloride hexa hydrate, nickel chloride, sodium borohydride, iron sulfate. Following chemicals were used as solvent: propanol, acetone, ethanol, kerosene oil. Analytical grade chemicals were purchased from SIGMA ALDRICH and utilized without any further purification.

### Sample preparation for different applications of Fe–Ni bimetallic nanoparticles in kerosene oil

#### Sample preparation for flash point and firepoint

First, in 100 ml of kerosene oil, we will prepare three different solution concentrations of 0 ppm, 30 ppm, 60 ppm and 90 ppm. Open tester cup is a tool used for the implementation of flash and fire point to obtain the distinct sample solution values to verify the nanocatalyst's impact on these temperatures. We check the impact of bimetallic nanoparticles on the kerosene fuel efficiency from these parameters.

#### Sample preparation for cloud point and pourpoint

The solutions of various concentrations (0 ppm, 30 ppm, 60 ppm, 90 ppm) were prepared for cloud and pour point by blending separate quantities of the nano catalyst sample (0, 0.003 g, 0.006 g, 0.009 g) with 100 ml kerosene gas. The impact of the nano catalyst was checked on the cloud point and the pour point of the pure and altered kerosene fuel.

#### Sample preparation for calorimeter

A bomb calorimeter tool is used to obtain the calorific values by evaluating the water produced by combustion responses. Prepare separate sample concentration *e.g.* 0 ppm, 10 ppm, 15 ppm and 20 ppm, using separate nano catalyst quantities such as 0, 0.0015 g, 0.003 g, 0.0045 g, in 50 ml of kerosene gas for this purpose.

#### Sample preparation for specific gravity

Prepare various sample (0, 0.0015 g, 0.003 g, 0.0045 g) with different concentrations (0 ppm, 30 ppm, 60 ppm, 90 ppm) in 50 ml of kerosene oil to obtain specific gravity values. The nanocatalyst's impact on the kerosene fuel's specific gravity will be verified. Specific gravity can be determined by the sample mass/volume in 50 ml kerosene oil.

#### Sample preparation for kinematic viscosity

Ostwald viscometer can be used to measure viscosity. Prepare various sample (0, 0.0015 g, 0.003 g, 0.0045 g) with different concentrations (0 ppm, 30 ppm, 60 ppm, 90 ppm) concentrations in 500 ml of kerosene oil to obtain viscosity values. The nanocatlyst's impact on the fuel's viscosity will be explored. From that parameter we conclude that with the tiny quantity of the bimetallic nanocatalyst, how much fuel effectiveness improved.

#### Sample preparation of degradation of methylene blue dye

The ratio for the preparation of solution of dye for different concentration is given below in Table 1 whereas Table 2 shows the ratios of solution of dye with different concentration of catalyst.

**Table 1:** Solution of dye for different concentration

Dye concentration	Dye solution	Water
10 ppm	10 ml	90 ml
15 ppm	15 ml	85 ml
20 ppm	20 ml	80 ml

**Table 2:** Solution of dye varying concentration of catalyst

Dye concentration	Catalyst concentration	H_2_O_2_ concentration
20 ppm	0.20 mg mL^−1^	2 ml
20 ppm	0.35 mg mL^−1^	2 ml
20 ppm	0.50 mg mL^−1^	2 ml

Dye concentration and H_2_O_2_ concentration remain same but catalyst concentration varies in all solutions as shown in Table 3.

**Table 3:** Solution of dye varying H_2_O_2_ concentration^a^

Dye concentration	Catalyst concentration	H_2_O_2_ concentration
20 ppm	0.20 mg mL^−1^	1 ml
20 ppm	0.20 mg mL^−1^	2 ml
20 ppm	0.20 mg mL^−1^	3 ml

a Dye concentration and catalyst concentration remain same but H_2_O_2_ concentration varies in all solution.

### Preparation of iron–zinc bimetallic nanoparticles

In the present study work for the synthesis of Fe/Ni bimetallic nanoparticles, the methods used to prepare the nanoparticles in the past were expensive and very complicated to handle thus the wet chemical method. Using the technique of moist chemical synthesis, preparation of iron–nickel bimetallic nanoparticles was produced, and iron oxide and nickel nitrate salts were used in it. Ferric oxide (FeCl_3_·6H_2_O) was drawn in a beaker and blended in 100 ml of ethanol/water (30/70) to 100 ml of distilled water with soluble and sodium borohydride (NaBH_4_). To remove surplus sodium borohydride, let the material acquired centrifuge well with distilled water. To achieve bimetallic nanoparticles, 50 ml ethanol solution has now been introduced to disperse the newly synthesized iron nanoparticles. Nickel nitrate (Ni(NO_3_)_2_) was then added mixed in 50 ml ethanol solution and well stirred for 30 minutes. After the reactions the particles were washed by distilled water and ethanol solution. After the reactions, the distilled water and ethanol solution washed the particles. The product is derided in the oven at 120 °C for 24 hours after washing.

### Instrumentation

UV-visible spectrophotometer (SP-300) was used to measure the maximum dye solution absorbance of various concentrations within the 400–800 nm range. The spectrophotometer used to check the efficacy of the bimetallic nanoparticles to degrade the organic colors. To know the crystal arrangements and geometry of the bimetallic nanoparticles, the samples were analyzed by NTU (National Textile University of Faisalabad) X-ray diffraction. The X-ray diffraction provides us with complete information on bimetallic nanoparticles' structural arrangements and geometry. Bimetallic nanoparticles (NPs) have been analyzed for morphology of Fe/Ni by scanning electron microscopy from NTU (Faisalabad National Textile University). The SEM images provide us with information on the surface morphology of bimetallic nanoparticles. The size, shape, geometry, chemical structure and morphology of bimetallic Fe/Ni nanoparticles were checked using NIBGE (National Institute of Biotechnology and Genetic Engineering) transmission electron microscopy. The TEM images provide us with a clearer view of the bimetallic nanoparticles' morphology compared to the SEM images.

### Statistical analysis

Linear regression was utilized to conduct the statistical analysis using all the data. The regression models were employed to assess the connection among various characterization parameters.

## Results and discussion

Iron and nickel salts have been used in the production of bimetallic Fe/Ni nanoparticles. Using different techniques, the synthesized bimetallic Fe/Ni nanoparticles were analyzed. Finally, various catalytic activity characteristics of iron–zinc bimetallic nanoparticles were carried out and experimental results were obtained.

### X-ray diffraction (XRD) analysis

**Fig. 1:**
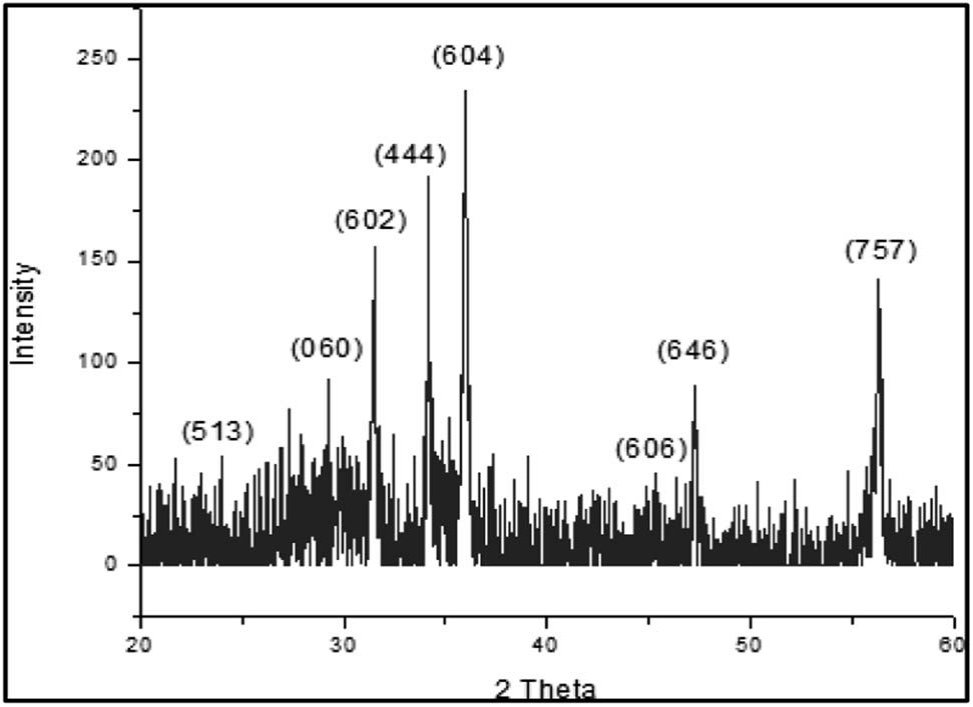
The XRD pattern of synthesized.

Fig. 1 shows the XRD pattern of the bimetallic nanocatalyst Fe/Ni newly prepared. The XRD findings provide us with complete information on the nanoparticles structure. The peaks are observed at 30.40°, 30.80°, 32.50°, 34.60°, 37.00°, 43.70°, 48.50° and 56.80° 2 with the corresponding miller indices (513), (060), (444), (604), (606), (646) and (757) respectively. The nanoparticles indicate the sharp peaks in the XRD pattern that obviously demonstrate that the item is structurally solely crystalline. The product's crystalline structure geometry is checked from the XRD pattern. These peaks also represent that these nanoparticles are made up of cubic unit cells at different positions. These peaks also check that the product is very pure and the product does not contain any impurities. The XRD pattern should have an additional peak for the impurities. The expansion of peaks also clearly shows that the product is within the range of nanometers and the size of most particles is below 100 nm.

**Fig. 2:**
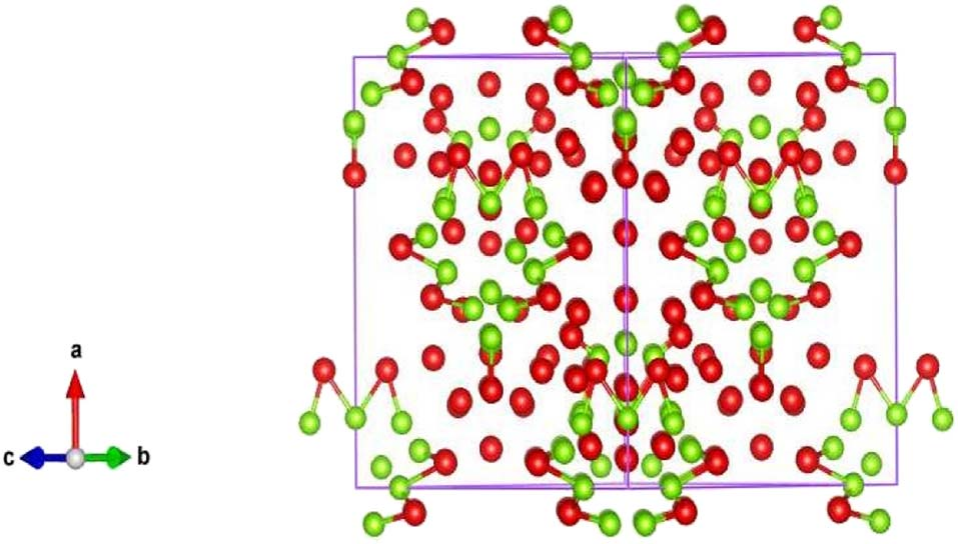
Cubic structure of Fe/Ni bimetallic nanoparticles.

The cubic structure indicates that the molecular formula Fe12 Ni36 has bimetallic nanoparticles of iron and nickel as shown in Fig. 2. These bimetallic nanoparticles' empirical formula is Fe1 Ni3. The XRD study also revealed that there is a cubic unit cell in the bimetallic nanoparticles. The iron-nickel bond ratio is 1 : 3 and the iron–nickel atom bond range is 2.45923 Å and the iron-nickel atom bond angle is 135.2245°. For the polyhedral structure of the bimetallic nanoparticles Fe/Ni, the two different bond angels are observed. The bond distance at the bottom of the bimetallic structure of Fe/Ni is 2.56865 Å and the bond distance at the center of the cubic structure is 2.75567 Å as shown in Table 4. At the center of the structure, the bond distance is greater than at the edge of the structure. The dihedral angle discovered in the bimetallic nanoparticles Fe/Ni structure is 64.8645°. From the figure it is observed that the 535 has 225 bond numbers in the polyhedral structure of the bimetallic Fe/Ni nanoparticles in the unit cell. The XRD assessment shows the cubic geometry of the bimetallic Fe/Ni nanoparticles. In several arrangements with completely distinct symmetry, the cubic structures are also achieved. The XRD assessment is therefore very essential and plays an important part in the advancement of nanotechnology because the XRD findings are very helpful about the geometry and shape of the nanoparticles.

**Table 4:** The crystal data of Fe/Ni bimetallic nanoparticles obtained from XRD analysis

Parameters	Iron–nickel bimetallic product
Name	Iron–nickel bimetallic nanoparticles
Chemical formula	Fe12Ni39
Crystal system	Face centered cubic
Type of unit cell	*F*4̄3*m* (216)
Parameters of the cell	
*A*	17.965 Å
*B*	17.965 Å
*C*	17.965 Å
*A*	90°
*B*	90°
*Γ*	90°
Volume of the one unit cell	5786.3 (Å)^3^
Number of distinct element	2
Calculated density of the product	7.37700 g cm^−3^
*x*, *y*, *z* atomic coordinate of the Fe atoms	0.602, 0.602, 0.602
*x*, *y*, *z* atomic coordinate of the Ni atoms	0.324, 0.324, 0.324
Bond angles	135.2245°
Bond lengths	2.45295 Å

### Scanning electron microscopy

**Fig. 3:**
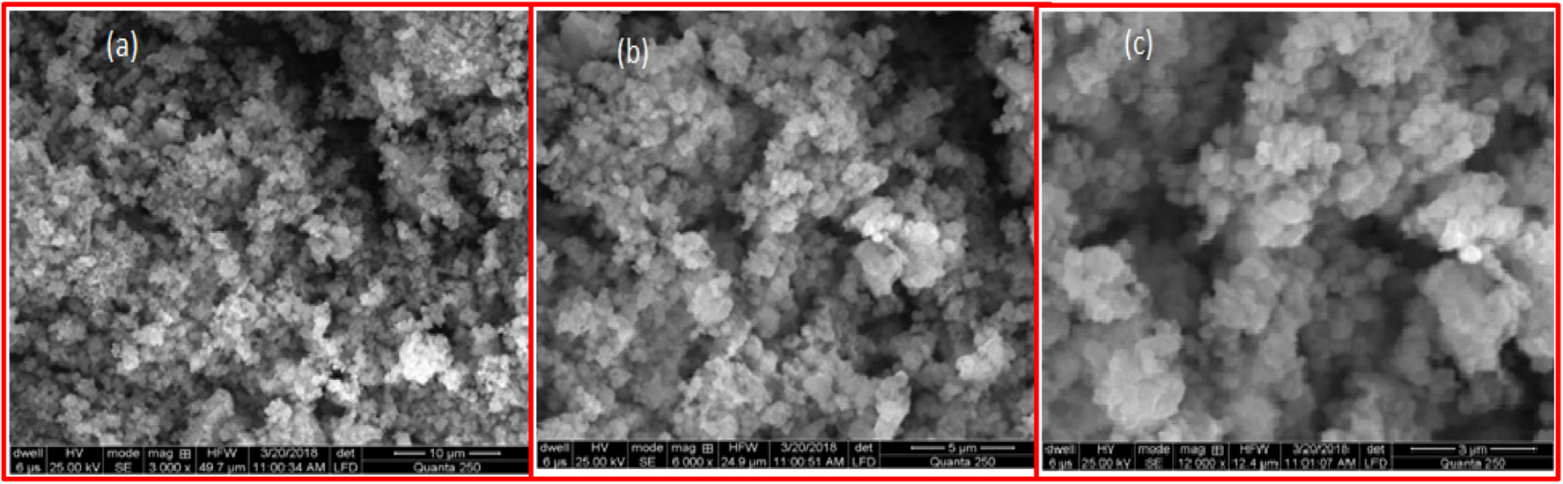
SEM images of newly synthesized Fe/Ni bimetallic nanoparticles at different ranges from (a) to (c).

The findings of Scanning Electron Microscopy (SEM) inform us about the nanoparticles' size, morphology, shape and porosity. Fig. 3 illustrates the SEM pictures of the bimetallic Fe/Ni nanoparticles at different magnifications. Fig. 3a and b SEM outcomes show that the nanoparticles have merged surfaces and that some nanoparticles are mostly irregular in form and size at 50 μm range. Fig. 3c shows that some nanoparticles are within 100 nm range. The rough edges of the nanoparticles are noticeable at elevated magnification in Fig. 3c. The adhesion of the particles of bimetallic nanoparticles to each other occurs owing to the attraction forces of wandering wall among themselves. Because of this, the aggregates in the pictures are very evident.

Fig. 3c indicates the product's large aggregates, and the bimetallic nanoparticles are present on these aggregates' surfaces. The very compact shape of the bimetallic nanoparticles. The shape of the bimetallic nanoparticles is uncertain and irregular.

### Transmission electron microscopy (TEM) observations

**Fig. 4:**
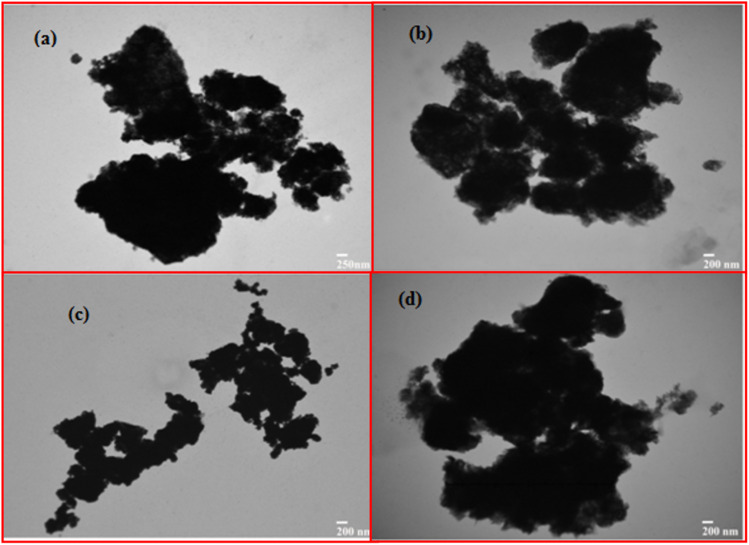
TEM images of newly synthesized Fe/Ni bimetallic nanoparticles with different aggregates from (a) to (d).

Transmission electron microscopy (TEM) offers us with helpful data about the nanoparticles' form, atomic size and chemical structure. Fig. 4 shows TEM pictures of bimetallic nanoparticles with multiple magnifications. Fig. 4 demonstrates that these nanoparticles vary in size and that most nanoparticles are irregular in form. Fig. 4 obviously shows the roughness of the nanoparticles at the corners. Fig. 4 represents more than 100 nm in the size of most nanoparticles. Also observed are particles of less than 100 nm size. The size of the nanoparticles in the nanometer range was noted at different magnifications. Various variables such as preparation techniques, structure, reduction agent, temperature, pH and time can affect the size of bimetallic nanoparticles. The TEM pictures show the rough nesses of the surface of these nanoparticles. These particles' hollow morphology is obviously noticeable from the dark colors of the light.

### Applications of Fe/Ni bimetallic nanoparticles as fuel additive

Fe/Ni bimetallic nanoparticles are used as fuel additives vastly. Fe–Ni BMNPs act as combustion catalysts, promoting more complete fuel oxidation, and reducing the formation of harmful carbonaceous residues. Their small size and high surface area facilitate better dispersion and interaction with the fuel, enhancing their catalytic effects. Additionally, their inherent magnetic properties may aid in fuel atomization and mixing, further improving combustion efficiency. By incorporating Fe–Ni BMNPs into fuel formulations, it is possible to achieve cleaner and more efficient combustion processes, thereby contributing to reduced emissions and environmental impact.

To verify its effectiveness, the fuel's combustion and physical characteristics are very crucial. The elevated octane number kerosene gas was used as a reference standard to verify the effectiveness of the modified kerosene gas. The impact of distinct bimetallic nanocatalyst concentration was explored on the distinct characteristics of kerosene fuel due to the use of kerosene fuel in separate kinds of heavy motors. By calculating the values of flash point, fire point, pour point, cloud point and calorific values, the combustion characteristics and properties of the pure kerosene fuel and the additive with different dosage of the bimetallic nanocatalyst are analysed. The particular gravity and viscosity are evaluated to study the physical characteristics of the natural kerosene gas and the additive with distinct dosage of the bimetallic nanocatalyst. These parameters were studied by the bimetallic nanocatalyst at different concentrations (0 ppm, 30 ppm, 60 ppm and 90 ppm). All findings achieved using separate bimetallic nanocatalyst concentrations were compared with the sample of pure kerosene gas.

## Combustion characteristics

### Flash point and fire point

**Fig. 5:**
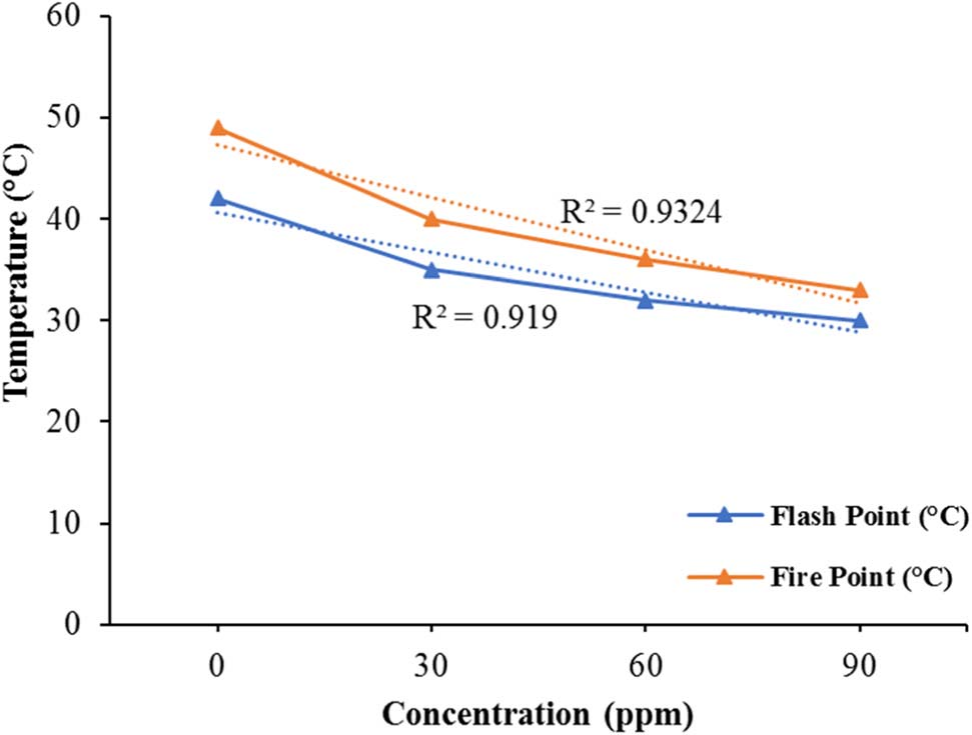
Effect of Fe/Ni bimetallic nanoparticles of fire point and flash point of kerosene oil.

The results of the linear regression analysis indicated a well-distributed data pattern along a straight line, as illustrated in Fig. 5 (*R*^2^ = 0.9324) for the fire point and (*R*^2^ = 0.919) for the flash point. These figures demonstrate the influence of varying nanocatalyst concentrations on both flame point and flash point. The point of flash is earlier than the point of fire. The fuel becomes ignitable with the air at the flash stage. It is evident from graph that in the lack of bimetallic nanoparticles, the flash point and fire point values of kerosene oil are very high. But the values of flash point and fire point decrease significantly by incorporating the nanocatalyst in the solution. As the concentration of the bimetallic nanocatalyst increases, the flash point and fire point values decrease directly as shown in Graph. Thus, by adding the very small amount of bimetallic nanocatalyst, the kerosene oil volatility increases. This variation in the flash point and fire point shows that bimetallic Fe/Ni nanoparticles can be used efficiently as catalysts and therefore have a very powerful capacity to improve kerosene fuel efficiency. Compared to prior work reported by Sajith *et al.*,^53^ on kerosene fuel effectiveness, the variation in the flash point and flame point is much greater. The newly synthesized Fe/Ni bimetallic nanocatalst is therefore very useful in improving the kerosene fuel efficiency.

### Physical characteristics

#### Cloud point and Pore point

The experimental data was fitted into a quadratic equation of the second order. The regression analysis indicated a satisfactory distribution of the data with (*R*^2^ = 0.8364) for cloud point and (*R*^2^ = 0.8364). In comparison to the additive kerosene fuel, the pour point and cloud point values of pure kerosene fuel are relatively elevated at certain levels.

**Fig. 6:**
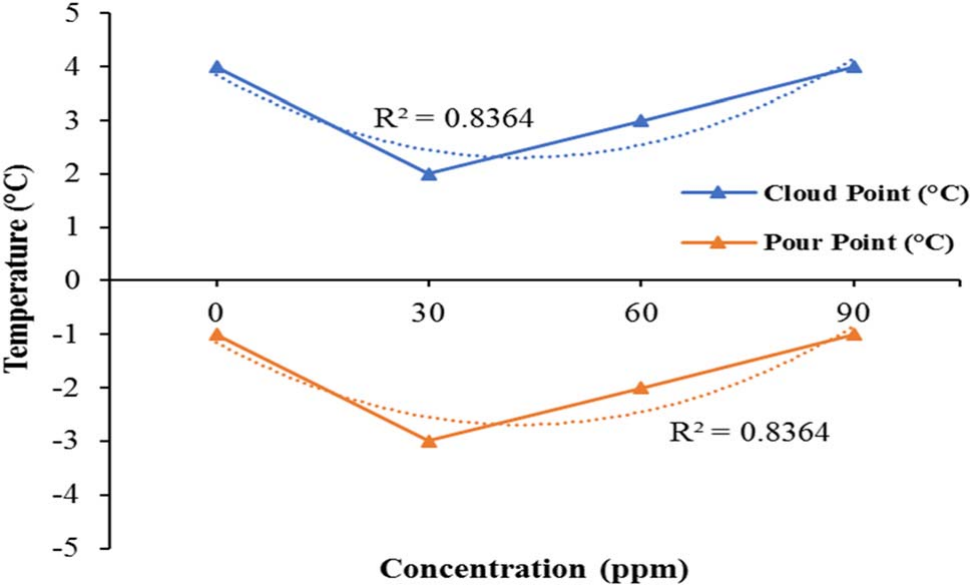
Effect of Fe/Ni bimetallic nanoparticles of pour point and cloud point of kerosene oil.

The cloud point and pour point values first slightly decrease and then start to increase as the bimetallic nanocatalyst's concentration increases. But the cloud point variation and the point of pouring when adding bimetallic nanocatalyst is not very important Fig. 6.

There is very little difference from pure kerosene fuel in the values of pour point and cloud point. The low temperature features of the additive kerosene fuel, such as cloud point and flame point, have not been significantly impacted. As shown in Fig. 8. Consequently, the bimetallic nanocatalyst has no significant.

### Kinematic viscosity

**Fig. 7:**
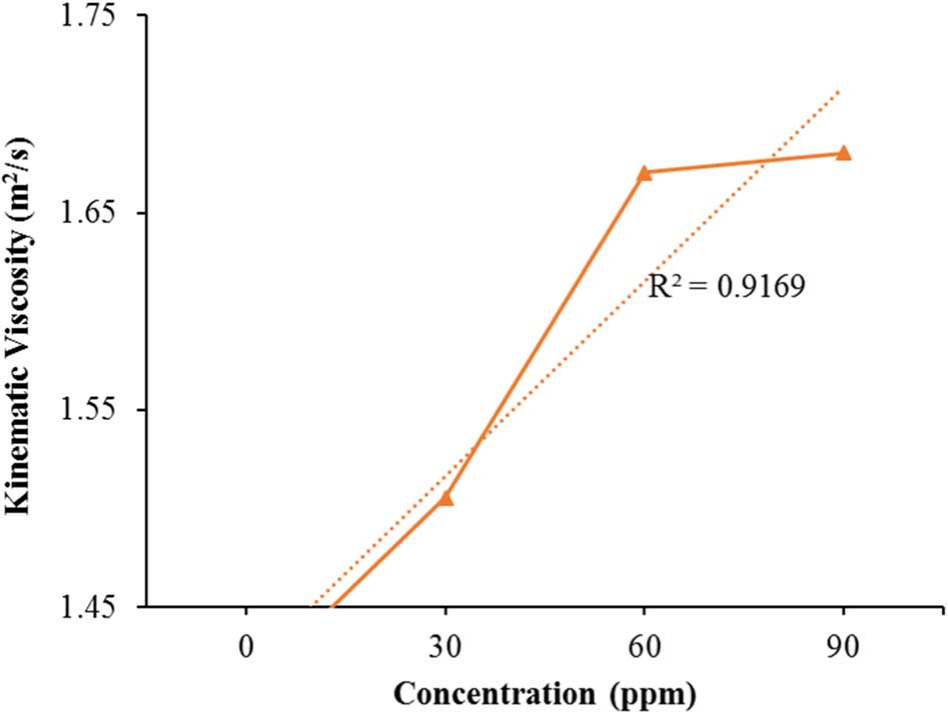
Effect of Fe/Ni bimetallic nanoparticles on the kinematic viscosity of kerosene fuel.

The experimental data underwent linear regression analysis. The graph in Fig. 7 illustrates the proper alignment of the data along a linear pattern, indicated by (*R*^2^ = 0.9169).

The viscosity of the blend kerosene oil and the additive kerosene nanoparticles measured using a viscosity meter. All samples' viscosity was evaluated at room temperature. The pure kerosene fuel's viscosity is 1.4078 m^2^ s^−1^. The viscosity of the pure kerosene gas and the nanocatalyst with distinct concentrations. Kerosene fuel viscosity rises linearly by raising nanocatalyst concentration. Fig. 7 plots the viscosity against distinct concentrations Table 4 shows that the viscosity of natural kerosene improves significantly by raising the Fe/Ni bimetallic nanocatalyst concentration. The sample with a big nanocatalyst concentration has a much higher viscosity value. Small quantity of the nanocatalyst is therefore very efficient in increasing the fuel's viscosity. Engine efficiency can be enhanced by raising the fuel's viscosity. Fe/Ni bimetallic nanocatalysts are therefore very helpful in increasing the fuel efficiency. The more viscous fuel offers more lubrication and the turbulent flow is therefore reduced. The flow rate of the modified kerosene fuel decreased compared to the pure kerosene fuel showing that the modified fuel is more viscous from the pure kerosene fuel and increased the resistance between the different layers and decreased the turbulence in the fuel flow. Thus the engine's effectiveness improves substantially. Thus the very tiny quantity of bimetallic nanoparticles is very helpful to boost the viscosity of the kerosene fuel and significantly boost its effectiveness.

### Specific gravity

**Fig. 8:**
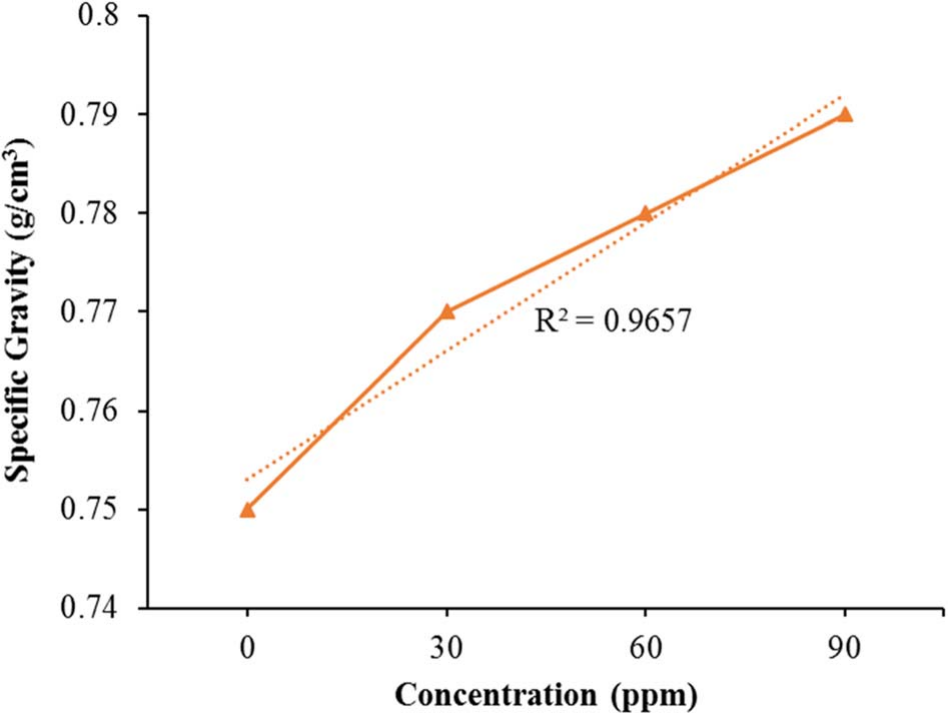
Effect of Fe/Ni bimetallic nanoparticles on the specific gravity of kerosene fuel.

The experimental data was incorporated into a quadratic equation of the second order, and the regression analysis demonstrated its appropriate distribution with (*R*^2^ = 0.9657). The determination of specific gravity for both pure kerosene oil and modified kerosene oil was carried out using a specialized gravity meter, as illustrated in Fig. 8 shows that the pure kerosene oil's particular gravity is small compared to the additive fuel. Specific gravity values improve by raising the bimetallic nanocatalyst concentration. The concentration of 90 ppm indicates peak specific gravity from the other reduced levels showing the impact of the nanocatalyst's tiny quantity on the specific gravity of the kerosene fuel. The engine's effectiveness can be enhanced by increasing the specific gravity of the fuel. The tiny dosage of bimetallic Fe/Ni nanocatalyst is thus very helpful to raise the specific gravity of the gas and thus greatly enhance the effectiveness of the kerosene fuel.

### Calorimetric values

**Fig. 9:**
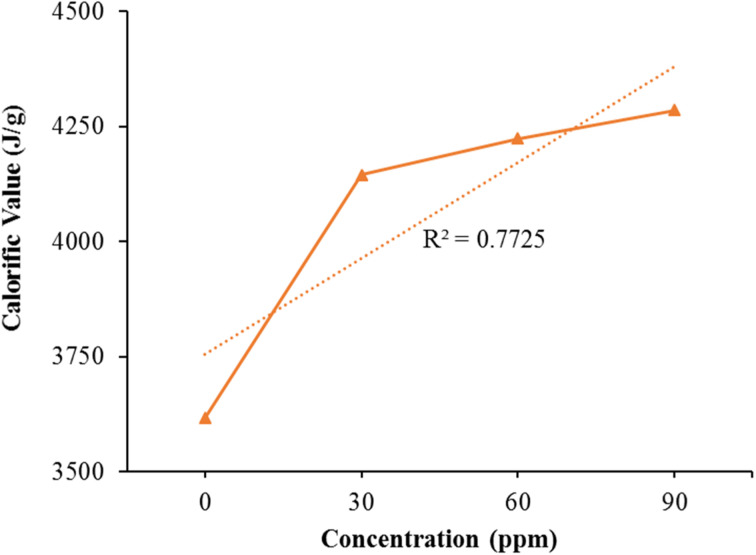
The calorimetric values of natural kerosene oil and the distinct dosage of the Fe/Ni bimetallic nanocatalyst.

Fig. 9 presents the calorimetric results for both untreated natural kerosene oil and varying dosages of the Fe/Ni bimetallic nanocatalyst. In comparison to the modified kerosene variant, the calorific value of pure kerosene oil is particularly lower. The data's proper distribution along a linear trajectory was confirmed through linear regression analysis, yielding an *R*^2^ value of 0.7725.

The calorific values improve linearly by raising the bimetallic nanocatalyst concentration. It is therefore evident from Fig. 9, that the bimetallic nanoparticles have a powerful impact on the kerosene fuel calorific values. The calorific values of pure kerosene fuel and altered fuel differ significantly. The sample with elevated bimetallic nanoparticles concentration has elevated calorific values. Bimetallic nanoparticles thus considerably increased the effectiveness of the kerosene fuel.

### Catalytic applications: catalytic degradation of methylene blue dye

Photocatalysis is a process where a catalyst facilitates a photochemical reaction upon exposure to light. Fe–Ni BMNPs possess unique properties that contribute to their enhanced photo-catalytic performance. The bandgap energy of these nanoparticles plays a crucial role in determining their ability to absorb light in the visible spectrum. Fe–Ni BMNPs with a narrower bandgap can harness a broader range of sunlight, making them more efficient photo-catalysts. To further improve their light absorption and electron–hole pair generation, Fe–Ni BMNPs may undergo surface modifications or possess functional groups. These modifications enhance the nanoparticles' light absorption capacity and create more active sites for photocatalytic reactions, leading to enhanced efficiency in generating electron–hole pairs during the photochemical process. The Fe–Ni bimetallic composition also significantly influences the photo-catalytic activity of these nanoparticles. The synergistic effect between iron and nickel results in unique electronic and catalytic properties. This synergistic effect enhances the overall efficiency of photo-catalytic reactions by promoting charge separation and improving the redox capabilities of the Fe–Ni BMNPs. Consequently, these bimetallic nanoparticles can more effectively initiate and drive photochemical reactions, making them highly promising candidates for various photocatalytic applications.

In aqueous solution, the methylene blue color is very stable. It stays active for a longer period of time in the water solution. Due to persistence in aqueous solution and toxicity, the methylene blue dye creates serious environmental effects. The methylene blue coloring is used to color the fabric's wide range and poses a severe environmental danger. The removal of methylene blue from the water solution is therefore of excellent interest to the researcher. Using the photo degradation method, the newly prepared nanoparticles were used to remove it from the waste water and thus studied the nanoparticles' catalytic effectiveness. In the presence of sunlight, the methyl blue color will be totally degraded within 30 minutes using catalyst and H_2_O_2_. The bimetallic nanocatalyst Fe/Ni is very efficient in very short time degrading the blue methylene dye.

The methylene blue dye's catalytic degradation was explored using the spectrophotometer U-2900UV/VIS. The solution 10 ppm, 15 ppm and 20 ppm strongly absorb 2.317, 2.932 and 3.4123 at 663.5 nm. The dye solution's absorbance improves significantly by raising the dye concentration. The methylene blue dye degradation was investigated without the use of nanoparticles, but after a long time the strong absorbance at 663.5 nm did not work. This shows that without the use of catalyst, methylene blue dye degradation was not feasible. The dye's photo degradation using various quantities of catalyst and H_2_O_2_ follows the kinetics of the first order. The bimetallic nanocatalyst demonstrates to be very efficient in easily and very short-term degradation of the variety of organic colors compared to the ancient and conventional techniques, which are mostly very expensive.

### Dye dependent UV-vis spectra

**Fig. 10:**
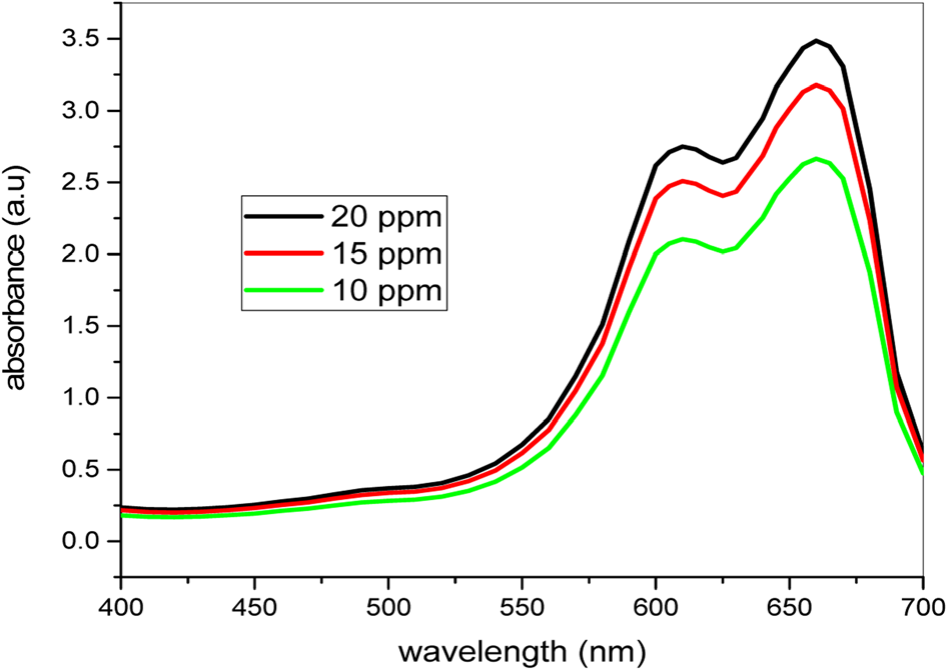
Comparison of dye absorbance at different concentrations in the absence of catalyst.

Fig. 10 shows the methylene blue UV-visible spectra with different levels of color solution without a catalyst. It is noted that the highest absorption of 10 ppm, 15 ppm and 20 ppm is 2.317, 2.932 and 3.412 at 663.5 nm. These values indicate that the absorption value improves considerably by raising the concentration of the dye. For the research of additional parameters using nanocatalyst and H_2_O_2_, the 20 ppm concentration was chosen. For this job, the elevated concentration of dye was selected because we have to face degradation of high concentration dye mostly in daily life. The large concentrations of these organic dyes are mostly present in the waste water of most of the industries. Most of the big levels of these organic colors are present in most industries' waste water.

### UV-vis spectra of degradation of dye with out catalyst

**Fig. 11:**
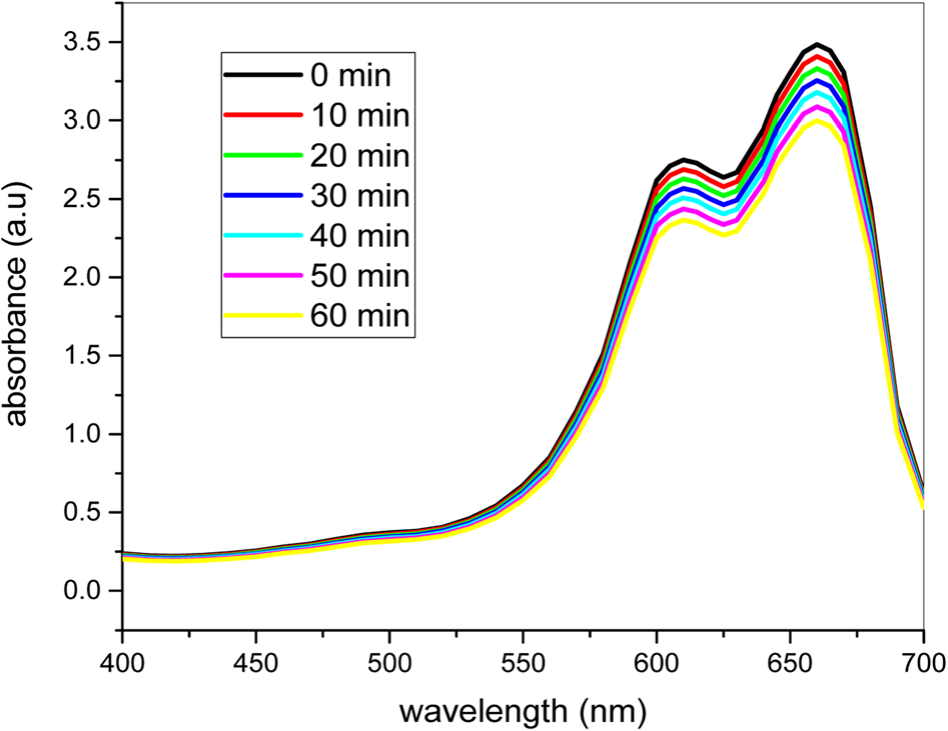
The degradation of the dye in the absence of catalyst at different time intervals under sunlight (conditions: dye = 20 ppm, [H_2_O_2_] = 2 ml).

Fig. 11 demonstrates the degradation of the product without a catalyst in the presence of 2 ml H_2_O_2_. Fig. 11 demonstrates the degradation of the product without a catalyst in the presence of 2 ml H_2_O_2_. The figure obviously demonstrates that the methylene blue dye degradation rate was very slow, and even after 60 minutes of stirring in sunlight, the dye did not degrade. The catalyst is therefore very important for the rapid degradation of the methylene blue dye. In 60 minutes, only 10–20% degradation was finished. Thus the color degradation without a catalyst is almost impossible. The methylene blue dye degraded to a very tiny extent with 2 ml of H_2_O_2_ and therefore the full degradation of the organic dyes is not only feasible with H_2_O_2_. The bimetallic nanocatalyst is very helpful for the degradation of organic coloring. These organic colors are commonly accessible in most industries' wastewater, and removal from wastewater is therefore very important.

### UV-vis spectra of degradation of dye with catalyst

**Fig. 12:**
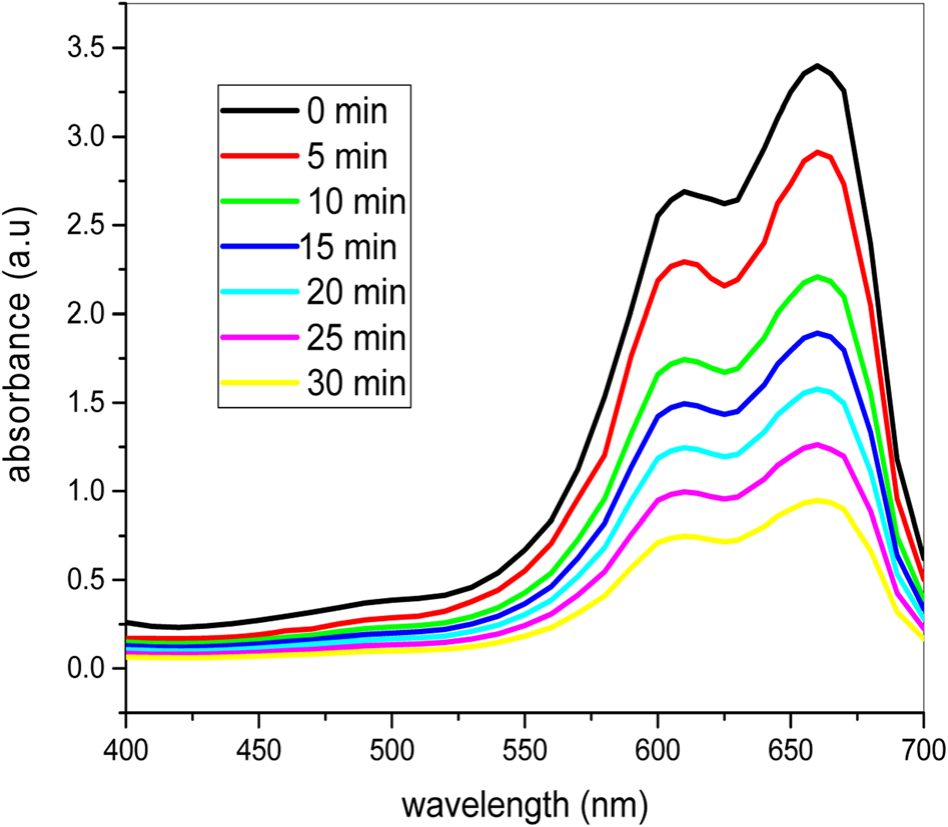
UV-vis spectra of the photocatalytic degradation of the dye at different time intervals under sunlight (conditions: dye = 20 ppm, [H_2_O_2_] = 2 ml, [catalyst] = 0.20 mg ml^−1^).

Fig. 12 shows how methylene blue dye degradation occurs over time with 0.20 mg ml^−1^ of nanocatalyst and 2 ml of H_2_O_2_ in sunlight. It is clear from the time-dependent UV-visible spectra that the methylene blue dye's maximum absorbance decreases with the passage of time consistently. At the beginning of the experiment at 663.5 nm the maximum absorbance was 3.412, but after 30 minutes in sunlight and stirring it consistently decreases to 0.91. Using catalyst and 2 ml H_2_O_2_ in sunlight, the methylene blue color is totally degraded after 30 minutes. Thus it is clear that the methylene blue dye is degraded completely and the bimetallic nanoparticles have great effect on the dye degradation process.

### Effect of catalyst concentration variation

**Fig. 13:**
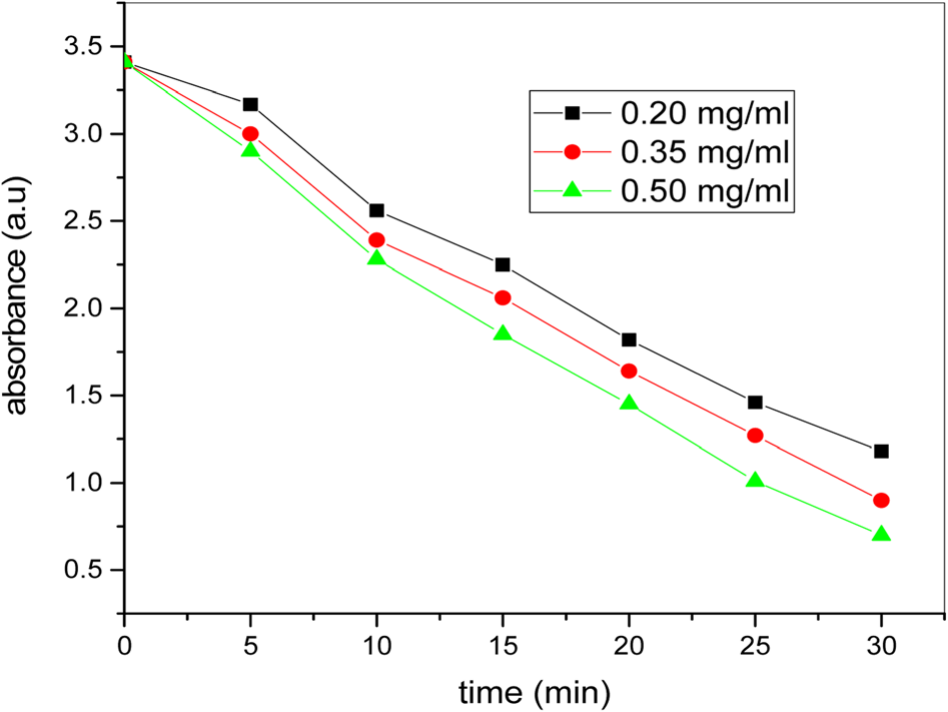
Plot of time *versus* absorbance for photocatalytic degradation of dye at different amount of catalyst (conditions: [dye] = 20 ppm, [H_2_O_2_] = 2 ml, [catalyst] = 0.20, 0.35 and 0.50 mg mL^−1^).

**Fig. 14:**
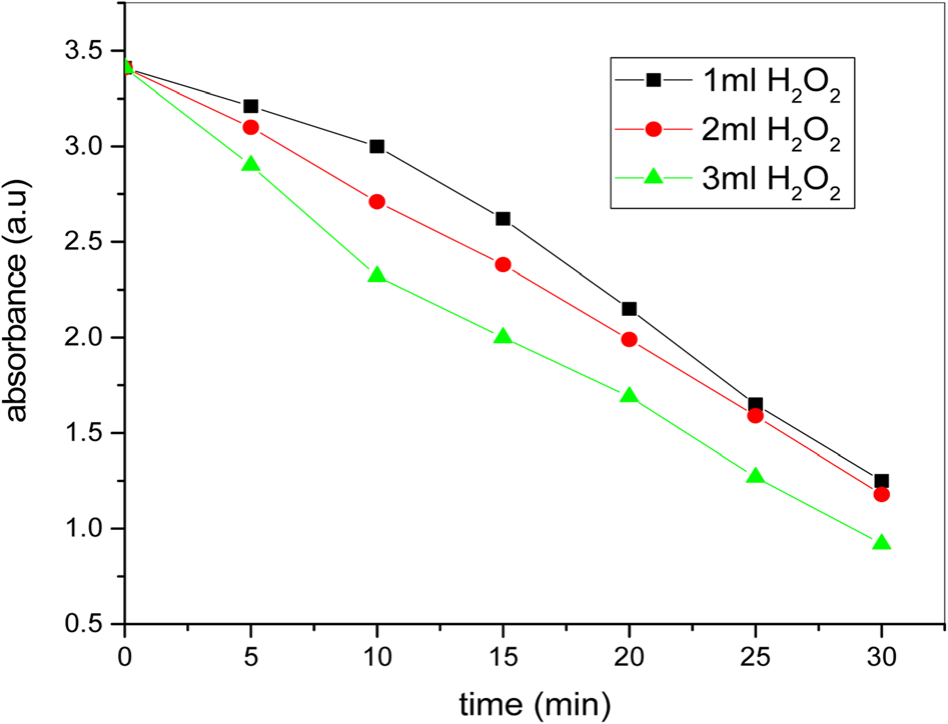
The plot of time *versus* absorbance for photocatalytic degradation of dye at different dosage of H_2_O_2_ (conditions: [dye] = 20 ppm, [H_2_O_2_] = 1 ml, 2 ml and 3 ml, [catalyst] = 0.20 mg ml^−1^).

Fig. 13 shows the plots of absorption of methylene blue dye *versus* time using catalyst dosage of 0.20, 0.35 and 0.50 mg ml^−1^ and 2 ml of H_2_O_2_ under sunlight. It is evident that absorbance reduces continuously over time for all the catalyst dosage, but at low catalyst dosage the degradation rate is small and rises as the dosage of the catalyst rises. From Fig. 14 it is thus clear that the degradation rate of the dye increases directly with the increase of the catalyst's dosage. The dye degraded in less time with a high amount of catalyst compared to the lesser amount of catalyst dosage. Therefore, with low catalyst dosage, catalyst velocity is very slow. The catalyst dosage of 0.50 mg ml^−1^ indicates sharp degradation in less moment, showing the significance of the dosage catalyst in methylene blue dye degradation. The very tiny quantity of the bimetallic Fe/Ni nanocatalyst is therefore very efficient for organic color degradation. The catalyst quantity differs in this entire degradation operation but the dye and H_2_O_2_ concentration constant.

### Effect of H_2_O_2_ concentration variation

Fig. 14 demonstrates time-related absorbance for distinct H_2_O_2_ amounts. In the event of 1 ml H_2_O_2_ concentration at the beginning there are no strong time-related decrees in the absorbance, but after 10 minutes the absorbance reduces immediately with time up to the degradation phase. But when the H_2_O_2_ level rises to 2 ml then the absorption reduces continuously from the starting point at the same pace until the point of degradation. In the event of the 3 ml H_2_O_2_, the absorbance from the start and the degradation of the dye is sharply decreased in shorter times compared to the other two levels. The picture catalytic degradation method stops after 30 minutes and the coloring is totally degraded. In the whole degradation process the amount of H_2_O_2_ varies but dosage of catalyst and concentration of dye remains same.

## Conclusions of the bimetallic nanoparticles act as catalysts for the application of dye degradation and fuel additive

The choice of iron and nickel for creating bimetallic nanoparticles was based on their favorable catalytic properties, widespread abundance, cost-effectiveness, compatibility with target applications, and unique physical characteristics. During the research process, various metal combinations may have been evaluated, but Fe–Ni BMNPs emerged as the most promising option, prompting their synthesis and subsequent investigation of their enhanced photo-catalytic and fuel additive properties. This rationale, along with the consideration of alternative metal combinations, provides readers with a clearer understanding of the selection process and highlights the novelty of Fe–Ni BMNPs as the chosen bimetallic nanoparticle system. It would be advantageous to include a discussion on the feasibility of scaling up the synthesis process for industrial applications. Additionally, the authors should address potential challenges that could arise during the scaling-up process. This discussion would provide valuable insights into the practical implementation of the synthesized Fe–Ni bimetallic nanoparticles and their potential impact in real-world applications.

Using hydrothermal technique, bimetallic Fe–Ni nanoparticles have been effectively synthesized. Using UV-visible spectroscopy, scanning electron (SE) microscopy, transmission electron (TE) microscopy and X-ray (XR) diffraction, these newly prepared bimetallic nanoparticles are researched. The product is pure, and X-ray (XR) diffraction assessment confirms the presence of hydrogen and oxygen atoms at the octaedron corner. The SEM findings indicate that there is a complete separation of the bimetallic nanoparticles and nanoscale particles. U-2900UV/VIS spectrophotometer was used to study methylene blue color degradation in the presence of bimetallic newly produced. At 663.5 nm, the blue methylene dye indicates peak absorption. The methyl blue dye degradation researched to learn about the photo catalytic conduct of the bimetallic nanoparticles that were newly synthesized. Methylene blue dye absorbance reduces from 3.412 to 0.92 by raising the dosage of bimetallic nanoparticles from 0.20 mg ml^−1^ to 0.50 mg ml^−1^. The dye's absorbance also reduces from 3.412 to 0.90 by raising the H_2_O_2_ from 1 ml to 3 ml in the presence of 0.20 mg ml^−1^ catalyst. The methylene blue dye's rapid degradation was noted with a catalyst dose of 0.50 mg ml^−1^ and a concentration of 3 ml H_2_O_2_. The impact of bimetallic nanoparticles on the effectiveness of kerosene diesel was also explored. Various parameters were conducted to evaluate the effectiveness of the modified fuel. Pure kerosene oil's calorific values increase by adding the dosage of bimetallic nanoparticles that proves the effective catalytic behavior of freshly prepared bimetallic nanoparticles. The pure kerosene oil's flash point and flame point temperature reduces considerably when the bimetallic nanoparticles are added. These bimetallic nanoparticles also have beneficial effects on the temperatures of the pour point and cloud point. Thus the pure kerosene oil's efficiency increases by adding the bimetallic nanoparticles dosage. These experimental results show that the freshly prepared bimetallic nanoparticles have very good photo catalytic properties and are very useful in improving the fuel efficiency.

## Conclusion

The nanoparticles are defined as particles with a length of at least 1 nm to 100 nm. The bimetallic nanoparticles are made up of two separate metallic elements with specific mixing arrangements, architecture, chemical structure and unique functions. Bimetallic nanoparticles have many advanced and beneficial properties because of the synergistic effect of the two metals combined. Compared to monometal nanoparticles, these bimetallic nanoparticles have significantly enhanced and helpful properties and have numerous unique applications in distinct areas. By changing morphologies and combining metals, the electrical, thermal, catalytic, magnetic, chemical and physical properties of bimetallic nanoparticles can be significantly enhanced. In the current research, the technique used to prepare iron–nickel bimetallic nanoparticles is very economical and environmentally friendly. The methylene blue dye degradation relies directly on the nanocatalyst concentration. Top degradation was observed using 2 ml of H_2_O_2_ and 0.50 mg ml^−1^ of dosage of bimetallic nanocatalyst. The newly prepared bimetallic nanocatalst is also used by different parameters to explore the effectiveness of the kerosene fuel. The fuel's calorific values improve significantly by adding a tiny quantity of the nanocatalyst, indicating that the nanocatalyst has a huge impact on obtaining energy from the fuel. By adding a tiny quantity of the nanocatalyst, the value of the flash point and fire point is significantly reduced. The nanocatalyst does not affect the cloud point and pour point to a large extent. The bimetallic nanocatalyst Fe/Ni therefore has very excellent catalytic characteristics.

## Data availability

It will be available online and can be provided on the reasonable request *via* email from the corresponding author.

## Author contributions

All authors contributed efficiently and dedicatedly in this manuscript and their credit to this manuscript is summarized as; Saba Jamil: conceptualization, validation, visualization, formal analysis, writing – review & editing. Shanza Rauf Khan: validation, visualization, formal analysis, writing – original draft revision. Shamsa Bibi: visualization, validation, writing – review & editing. Nazish Jahan: methodology, data curation, review & editing. Nadia Mushtaq: visualization, validation, writing – review & editing. Faisal Rafaqat: formal analysis, visualization, validation, writing – review & editing. Rais Ahmad Khan: visualization, validation, writing – review & editing. Waqas Amber Gill: visualization, validation, writing – review & editing. Muhammad Ramzan Saeed Ashraf Janjua: project administration, supervision, writing – review & editing.

## Conflicts of interest

There was no known conflict among the authors.

## Supplementary Material
